# Phase II study of selumetinib, an orally active inhibitor of MEK1 and MEK2 kinases, in KRAS^G12R^-mutant pancreatic ductal adenocarcinoma

**DOI:** 10.1007/s10637-020-01044-8

**Published:** 2021-01-06

**Authors:** Cara Kenney, Tricia Kunst, Santhana Webb, Devisser Christina, Christy Arrowood, Seth M. Steinberg, Niharika B. Mettu, Edward J. Kim, Udo Rudloff

**Affiliations:** 1 Thoracic & GI Oncology Branch, Center for Cancer Research, National Cancer Institute, Bethesda, MD USA; 2 Division of Hematology and Oncology, UC Davis Comprehensive Cancer Center, Sacramento, CA USA; 3 Duke Cancer Institute – Oncology CRU, Duke University Medical Center, Durham, NC USA; 4 Biostatistics and Data Management Section (BDMS), Office of the Clinical Director, for Cancer Research, National Cancer Institute, Rockville, MD USA; 5 Rare Tumor Initiative, Pediatric Oncology Branch, Hatfield Center, Center for Cancer Research - 2B-34D, National Cancer Institute, 10 Center Drive, Bethesda, MD 20892-0001 USA

**Keywords:** Selumetinib, MEK inhibitor, Pancreas cancer, KRAS G12 mutational isoform, Phase II

## Abstract

*Background* Preclinical evidence has suggested that a subset of pancreatic cancers with the G12R mutational isoform of the KRAS oncogene is more sensitive to MAPK pathway blockade than pancreatic tumors with other KRAS isoforms. We conducted a biomarker-driven trial of selumetinib (KOSELUGO™; ARRY-142886), an orally active, allosteric mitogen-activated protein kinase 1 and 2 (MEK1/2) inhibitor, in pancreas cancer patients with somatic KRAS^G12R^ mutations. *Methods* In this two-stage, phase II study (NCT03040986) patients with advanced pancreas cancer harboring somatic KRAS^G12R^ variants who had received at least one standard-of-care systemic therapy regimen received 75 mg selumetinib orally twice a day until disease progression or unacceptable toxicity occurred. The primary outcome of the study was best objective response (BOR). *Results* From August 2017 to February 2018 a total of 8 patients with confirmed somatic KRAS^G12R^ mutations and a median age of 61.5 years were treated with selumetinib. Seven out of eight (87.5%) had received two or more lines of prior systemic chemotherapy. After a median follow-up period of 8.5 months (range 2 to 20), three patients had stable disease for more than 6 months while receiving selumetinib. No patients achieved an objective partial response. Median progression-free survival (PFS) was 3.0 months (95% CI, 0.8–8.2) and median overall survival (OS) 9 months (95% CI, 2.5–20.9). *Conclusion* This study in heavily pre-treated pancreatic adenocarcinoma patients suggests alternative strategies beyond single agent MEK inhibition are required for this unique, molecular subset of pancreatic cancer patients. The trial was registered on February 2nd, 2017 under identifier NCT03040986 with ClinicalTrials.gov.

## Introduction

There is a grave, unmet medical need for improved treatment options for patients afflicted by pancreas cancer. Five-year survival rates of less than 10% have not substantially changed over the last three decades. Pancreas cancer is expected to rank 2nd in cancer-related mortality in the U.S. by the year 2030 surpassing breast and colorectal cancer [1, 2].

There currently exists a lack of effective targeted therapies for pancreatic cancer patients. The anti-EGFR inhibitor erlotinib (Tarceva™) received FDA approval based on minimal gains in progression-free survival (PFS) and overall survival (OS) over single agent gemcitabine [3]. The PARP inhibitor olaparib (Lynparza™) is approved for maintenance treatment of metastatic pancreatic adenocarcinoma with germline BRCA mutations [4]. However, these variants are detected in approximately 5–7% of patients. Other therapies approved for even smaller molecular subgroups include the immune checkpoint inhibitor pembrolizumab in MSI-H, and the neurotrophic receptor tyrosine kinase (NTRK) inhibitor larotrectinib (Vitrakvi™) for NTRK gene fusion-positive tumors [5, 6].

The vast majority of pancreatic cancers (up to ≥94%) harbor mutations of the KRAS oncogene. There is ample preclinical evidence that KRAS mutations are essential drivers of pancreas cancer development and progression, govern the unique metabolomic and transcriptomic landscape, and are involved in pancreatic cancer stem cell formation as well as mediation of resistance to chemo- and molecular therapy [7, 8]. Thus, with the emergence of small molecule inhibitors targeting MEK, an integral enzyme of the RAS/RAF/MEK/ERK pathway, pancreatic cancer patients were actively accrued onto initial MEK inhibitor studies including trials with selumetinib [9, 10]. Unfortunately, randomized phase II studies of selumetinib in the 2nd-line setting either as a single agent compared to oral capecitabine, or in combination with the AKT inhibitor MK-2206 compared to oxaliplatin and 5-flourouracil, failed to demonstrate benefit of selumetinib over chemotherapy [11, 12]. These findings are in line with results from other clinical studies with selumetinib in unselected patients with KRAS-mutated cancers which have been discontinued due to lack of efficacy [13, 14]. The observed lack of clinical activity also affirms preclinical findings on the significant heterogeneity of KRAS signaling output including effector signaling outside the canonical RAS/RAF/MEK/ERK cascade [15]. In search of a molecular classifier for pancreatic cancers vulnerable to MAPK pathway blockade via MEK inhibition which is not dependent on a complex gene expression signature, our prior work identified the KRAS mutational isoform G12R as a candidate [16]. KRAS mutations affecting codon 12 comprise of nearly all KRAS mutations in pancreas cancer [17]. KRAS G12R mutations are the third most common KRAS variant in pancreas cancer after KRAS G12D and G12V mutations and comprise up to 20% of KRAS mutations in some studies [17]. KRAS G12R mutations are highly unique for pancreas cancer as they are exceedingly rare, or non-existent, in other cancers (1% in thyroid cancers, <2% in NSCLC, <2% in colon cancers of all KRAS mutant cancers) [17]. The KRAS mutational isoform G12R fails to engage with a key effector, p110α PI3K (PI3Kα), which is associated with different metabolic regulation and pharmacological vulnerabilities including increased sensitivity to selumetinib in patient-derived xenotransplantation models [16]. KRAS mutational isoform status and KRAS mutational status per se (KRAS wild type versus KRAS mutant) are associated with different clinical outcomes including overall survival in pancreas cancer [8, 18, 19]. This phase II study aimed to test above preclinical observations in the clinic by evaluating the efficacy of selumetinib in pancreas cancer patients with confirmed somatic KRAS^G12R^ mutations who had received at least six months of prior systemic therapy.

## Patients and methods

### Patients

All patients had histologically confirmed locally advanced or metastatic pancreas cancer, received at least 6 months of 5-flourouracil- or gemcitabine-based treatments for pancreas cancer, and had measurable disease. Patients had Clinical Laboratory Improvement Amendments (CLIA)-confirmed somatic KRAS^G12R^ mutations as determined by sequence analysis of archival tumor sample or mandatory screening tumoral biopsy and matched normal DNA from any specimen obtained from the individual, Eastern Cooperative Oncology Group (ECOG) performance status ≤1 or Karnofsky index ≥70%, and normal organ and marrow function. Patients who received prior anti-EGFR kinase inhibitors, had known brain metastases, or had medical contraindications making administration of MEK inhibitors hazardous were excluded. The study was sponsored by NCIs Cancer Therapy Evaluation Program (CTEP), approved by the Central Institutional Review Board (CIRB) for the National Cancer Institute, registered with ClinicalTrials.gov (ClinicalTrials.gov Identifier: NCT03040986), and was conducted at participating centers of NCIs Experimental Therapeutics Clinical Trials Network (ETCTN).

### Evaluation of response and toxicity

Selumetinib was administered as an oral dose of selumetinib sulfate 75 mg twice daily (in the morning and evening) taken two hours after a meal and one hour before the next meal. Selumetinib was given continuously within treatment cycles, one cycle equaled 28 ± 2 days. Disease response assessment by CT scan was performed after cycle 1 and then every two cycles thereafter using standard Response Evaluation Criteria in Solid Tumors (RECIST) criteria version 1.1. Patients were taken off selumetinib treatment in case of disease progression or occurrence of adverse events which were graded by NCI Common Terminology Criteria for Adverse Events (CTCAE) criteria version 5.0. Dose adjustments were made following dose de-escalation recommendations for selumetinib to dose level minus one (50 mg twice daily) and dose level minus two (75 mg once daily) graded according to CTCAEv5.0.

### Role of sponsor

The study was sponsored by NCI CTEP. Selumetinib was provided by CTEP through a Cooperative Research and Development Agreement (CRADA) with AstraZeneca for this investigator-initiated clinical trial. Enrollment, treatment decisions, and all analyses were solely made by the clinical teams at the ETCTN centers without input from AstraZeneca.

### Statistical considerations

This open-label, non-randomized, multi-center phase II trial aimed to determine the best objective response (BOR) rate of selumetinib administered as 75 mg orally twice daily on a continuous schedule in patients with advanced pancreatic cancer harboring KRAS^G12R^ mutations within a Simon two-stage phase II design. All patients who received at least one cycle of selumetinib were evaluable. To determine whether selumetinib is associated with a response rate (PR + CR) that can rule out 5% (p0 = 0.05) in favor of an improved response rate of 30% (p1 = 0.30), and using alpha = 0.10 (probability of accepting a poor agent) and beta = 0.10 (probability of rejecting a good agent), initially 7 evaluable patients were planned to be enrolled. If 0 of 7 patients responded, then no further patients were planned to be enrolled. If 1 or more of the first 7 evaluable patients enrolled had a clinical response, then accrual will continue until a total of 21 evaluable patients have been enrolled. If one or two patients of the 21 had a clinical response, this was considered inadequate for further investigation of this regimen, if ≥3 responded, then this will warrant further investigation in a subsequent trial. Under the null hypothesis (5% response rate), the probability of early termination was calculated as 70%. Analysis of secondary endpoints included PFS and toxicity.

## Results

### Patient characteristics

In total, eight patients were enrolled (Table 1). The median age was 61.5 years (range 49–72). Patients were heavily pretreated. Seven out of eight (87.5%) had at least two lines of prior systemic chemotherapy, half of accrued patients had three or more lines of chemotherapy, and five out of eight patients (62.5%) had undergone prior surgery. Seven out of eight (87.5%) had at least two or more organs involved with metastases.

**Table 1:** Patients characteristics

	Number of patients (%)
Age (years)	
Mean (standard deviation)	61.25 (7.644)
Median (range)	61.5 (49–72)
Gender	
Male	4 (50)
Female	4 (50)
Race	
Caucasian	7 (87.5)
Non-Caucasian	1 (12.5)
Location of primary tumor	
Head / Body	5 (62.5)
Tail	3 (37.5)
Previous treatments	
Radiation	3 (37.5)
Surgery	5 (75)
Multiple agents systemic	8 (100)
Chemotherapy	
Number of previous lines of systemic chemotherapy	
1	1 (12.5)
2	3 (37.5)
≥3	4 (50)
Number of metastatic sites (involved organs)	
1	2 (25)
2	3 (37.5)
≥3	3 (37.5)
Mean (range) of pretreatment serum tumor markers	
CEA (ng/mL; range)	6.367 (1–23.8)
CA19–9 (U/mL; range)	1928 (6.9–8958)

### Response and survival

**Fig. 1:**
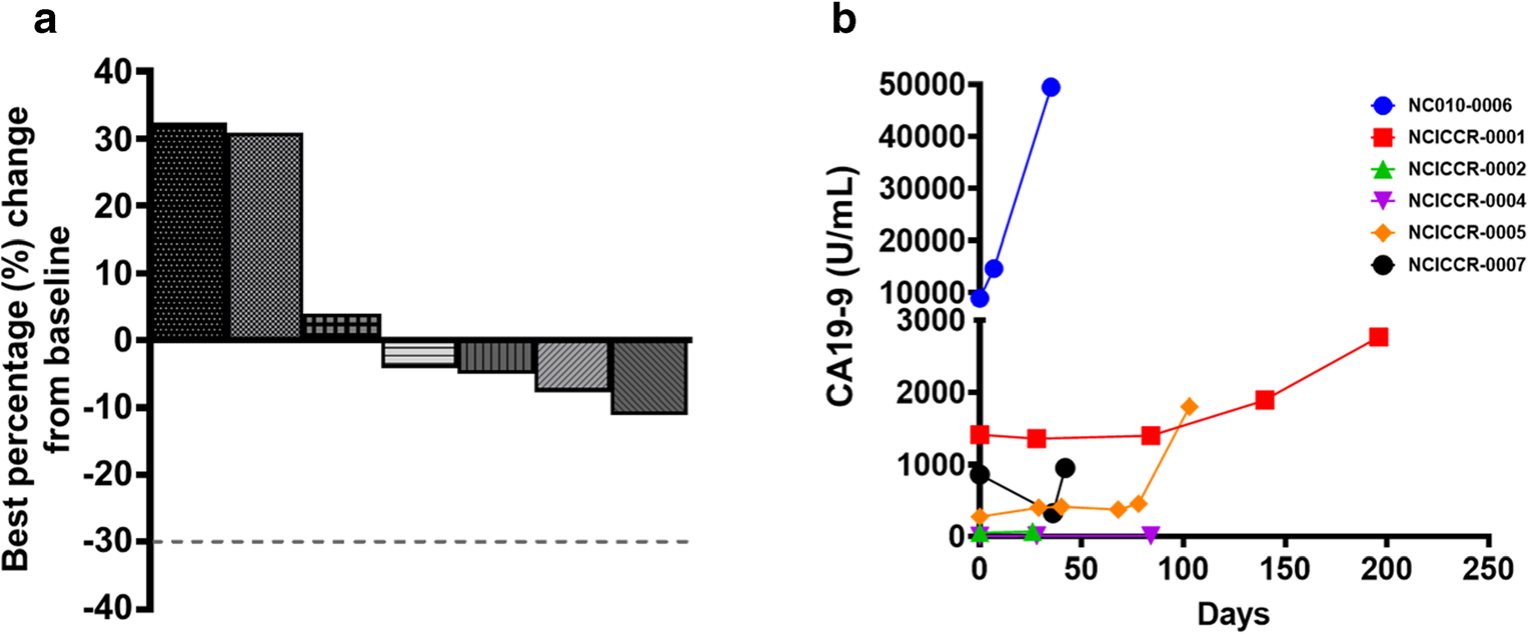
Clinical and biochemical responses of selumetinib in KRAS^G12R^-mutant pancreatic cancer patients. **a**. Best objective responses (BORs) measured as best percentage change in tumor volume by RECIST1.1 from baseline of patients treated with selumetnib. Dashed line indicates cut-off for partial response. **b**. Serum CA19–9 concentrations (U/mL) pre- and on-treatment with selumetinib

**Fig. 2:**
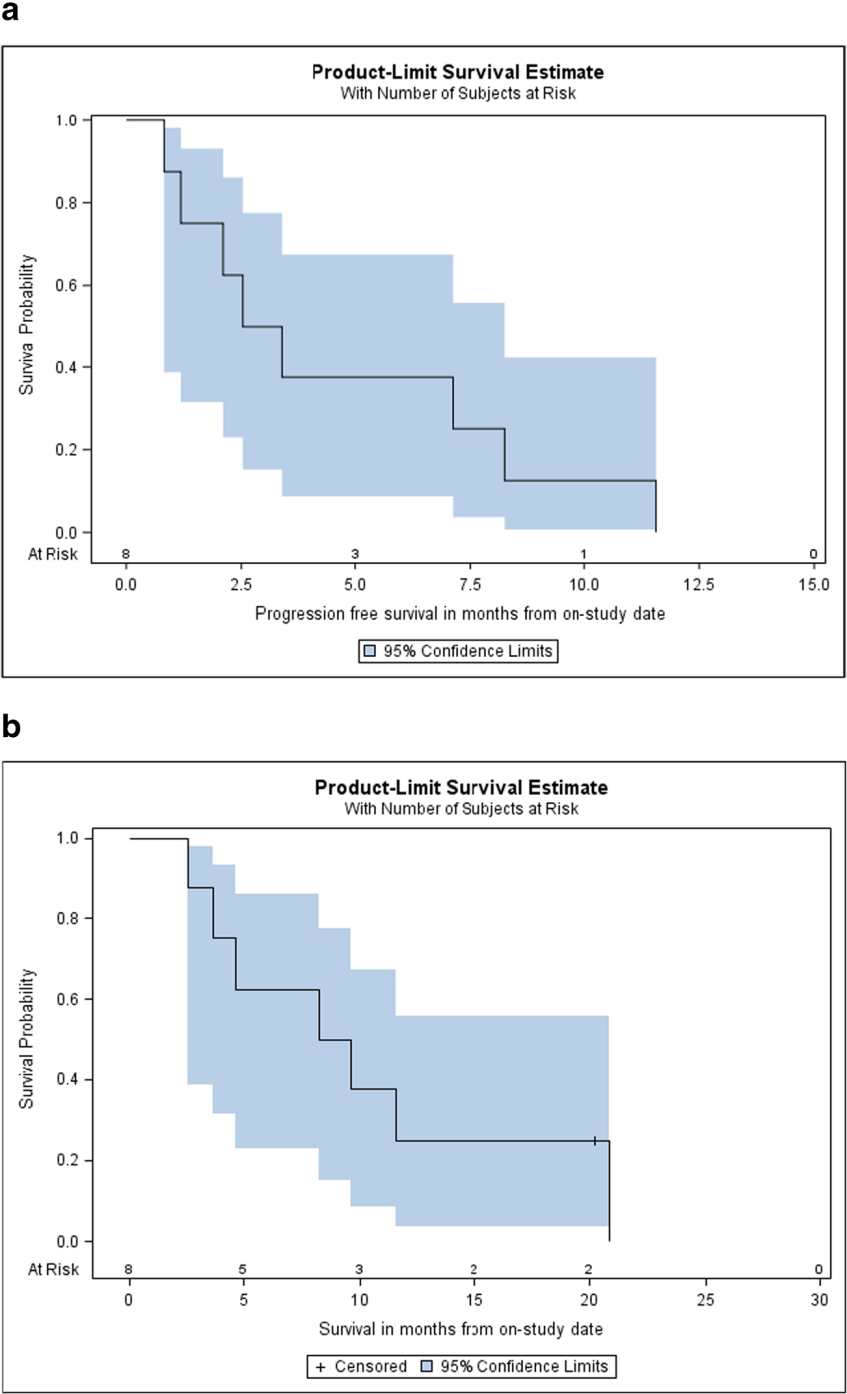
Survival outcomes with selumetinib. **a**. Kaplan-Meier estimate of progression-free survival (median PFS = 3.0 months (95% CI, 0.8–8.2 months). **b**. Kaplan-Meier estimate of overall survival (median OS = 8.9 months (95% CI, 2.5–20.9 months). Censored patients are indicated by -

There were no objective partial or complete responses by RECIST. In line with the pre-specified two-stage design the study did not move to the second stage and accrue additional patients (Fig. 1). Three patients had stable disease on selumetinib treatment for ≥6 months, and one of the three patients only experienced disease progression after ≥8 months after discontinuing selumetinib due to hepatic toxicity. There was one non-sustained biochemical response (≥50% reduction of tumor markers pre-treatment) at the end of cycle one in enrolled patients who had pre- and on-treatment CA19–9 levels available. During a median follow-up period of 8.5 months (range 2 to 20+) five patients experienced disease progression, one patient died, and two patients came off treatment due to adverse events, one patient due to treatment-related and one due to non-treatment related adverse events. Median PFS was 3.0 months (95% CI 0.8–8.2) and median overall survival (OS) 8.9 months (95% CI 2.5–20.9) (Fig. 2). Median duration of treatment was 3 months (range, 0.8–8.2).

### Adverse events

The toxicity profile of selumetinib was in line with previous reports in pancreas cancer. Grade 3 treatment-related adverse events included hepatic dysfunction, pancreatitis, hypertension, dyspnea, and heart failure as well as pancreatitis (Table 2). Permanent dose reductions to dose level minus one occurred in two (25%) patients with one leading to subsequent discontinuation of selumetinib due to hepatic toxicity.

**Table 2:** Treatment-related adverse events

	Grades 1–3 (%)	Grades 4–5 (%)
Increase in alanine aminotransferase	8 (100)	2 (25)
Increase in aspartate aminotransferase	8 (100)	1 (12.5)
Increase in alkaline phosphatase	3 (37.5)	–
Blood bilirubin increased	1 (12.5)	–
Abdominal pain	3 (37.5)	–
Abdominal distension	1 (12.5)	–
Bloating	2 (25)	–
Diarrhea	1 (12.5)	–
Nausea	3 (37.5)	–
Vomiting	2 (25)	–
Pancreatitis	1 (12.5)	2 (25)
Edema limbs	6 (75)	–
Edema trunk	1 (12.5)	–
Hypertension	6 (75)	2 (25)
Dyspnea	3 (37.5)	2 (25)
Heart failure	0 (0)	1 (12.5)
Anemia	3 (37.5)	–
Decrease lymphocyte count	2 (25)	–
Decrease neutrophil count	2 (25)	–
Rash acneiform	1 (12.5)	–
Rash maculo-papular	4 (50)	–

## Discussion

The KRAS^G12R^ mutational isoform is uniquely more prevalent in pancreas cancer compared to other KRAS-driven solid organ cancers and was previously shown to be a negative prognostic factor for overall survival compared to pancreatic cancers with KRAS G12D or G12V mutations [17, 18]. Preclinical studies identified a selective lack of RAS-driven macropinocytosis, altered metabolic regulation, and unique pharmacological vulnerabilities including increased responsiveness to selumetinib in the KRAS^G12R^ molecular subtype as rationale for clinical testing [16]. While in this phase II study the MEK inhibitor selumetinib did not induce any partial or complete responses, there were three patients with stable disease ≥6 months. The lack of more robust clinical activity in this study is, in part, in contrast to the recent role of MEK inhibitors in BRAF^V600^-mutant melanoma or selumetinib in neurofibromatosis type 1 inoperable plexiform tumors and may be attributed to several different factors [20]. Blockade of MAPK pathway signaling in BRAF-mutated melanoma or NF-1-mutant plexiform neurofibromas robustly induces cell death, whereas inhibition of MEK in KRAS-mutant pancreas cancer, including in KRAS^G12R^-mutant preclinical models, is inducing p27-dependent cell cycle arrest without inducing apoptosis [21]. Since tumor control via primarily cytostatic mechanism is more dependent on continuous exposure and target inhibition, drug treatments of solid organ tumors exerting a cytostatic mechanism have been cited as more vulnerable to pharmacological failure compared to agents inducing cell death [22]. Additionally, the emergence of adaptive resistance mechanisms in these genetically complex cancers might have contributed to the lack of objective clinical responses by selumetinib [23]. On the other hand, in light of the more aggressive natural history of tumors with the KRAS^G12R^ genotype it is tempting to speculate that the lack of disease progression for six months in three out of eight patients, in particular in the presence of a cytostatic mechanism of the investigational agent, may indicate an efficacy signal [18, 24].

Another important factor for the observed outcome of the study could have been the selected dose and the possible lack of sustained signaling inhibition. It is generally suggested that 80 % or greater suppression of phospho-ERK levels are required to induce tumor control [25]. Early phase I studies of selumetinib show effective (≥80%) phospho-ERK target inhibition in tumoral biopsies and peripheral blood lymphocytes at dose levels of 100 mg free selumetinib base suspension given twice a day or, in peripheral blood lymphocytes, with 75 mg hydrogen sulfate oral capsules administered twice a day [9, 26]. These reports were followed by a well-done tumoral PD study in colorectal cancer patients who received selumetinib and the AKT inhibitor MK-2206 and who underwent serial biopsies for quantitative measurements of p-ERK and p-AKT levels pre- and on-treatment [22]. Neither at the initial selumetinib sulfate dose of 75 mg once daily nor the increase dose of 100 mg once daily achieved the pre-specified p-ERK suppression of ≥70% suppression when comparing p-ERK levels on- to pre-treatment, baseline levels. Only a quarter of patients in this study showed a p-ERK level decrease exceeding 50% of baseline levels. Thus, with a plasma mean half-life of selumetinib of ~six hours and in the absence of tumoral PK measurements suggesting selumetinib accumulation upon multiple dosing, the administered dose of 75 mg twice a day in this study, while still 50% higher than the high dose level in the study of Do and colleagues, might have been insufficient for effective target suppression. At 75 mg selumetinib administered twice daily, in our study two out of the eight patients required dose reductions due to treatment-related hepatic toxicity, with one patient discontinuing selumetinib after six months of stable disease. These concerns for a narrow therapeutic window might be particularly relevant in pancreatic tumors which are characterized by a desmoplastic stroma creating a formidable barrier for effective drug penetration and drug delivery. The concern about target inhibition highlights also one of the major limitations of the study; serial tumor biopsies would have been required to confidently establish PK/PD relations upon selumetinib treatment and assess target engagement. Tumoral biopsies could have been also interrogated for possible resistance mechanisms of MEK inhibition. Determination of KRAS mutant allele frequencies (MAFs) on circulating free tumor DNA (ctDNA) of liquid biopsies might overcome the challenges of invasive tumoral sampling; however, a recent direct comparison of KRAS MAFs to standard serum CA19–9 levels in a large pancreas cancer patient cohort has not shown superiority in prognostication of the liquid biopsy technology [24]. While this phase II study of single agent MEK inhibition with selumetinib in KRAS^G12R^-mutant pancreatic tumors, which previously were reported to be associated with less favorable survival rates compared to other KRAS mutation, did not meet its primary endpoint, three patients had stable disease for more than six months. Future targeted strategies in this unique subgroup of pancreatic tumors should consider strategies like dual blockade of the MAPK pathway or combination therapies like MEK or ERK inhibition in combination with autophagy inhibition shown in preclinical testing to act synergistically [16].

## Data Availability

The datasets generated during and/or analyzed during the current study are available from the corresponding author on reasonable request.
